# ELDER ABUSE TRAINING NEEDS AMONG NURSING HOME SOCIAL SERVICES DIRECTORS

**DOI:** 10.1093/geroni/igac059.1566

**Published:** 2022-12-20

**Authors:** Amy Roberts, Georgia Anetzberger, Kevin Smith, Mercedes Bern-Klug

**Affiliations:** Miami University, Oxford, Ohio, United States; Case Western Reserve University, Cleveland, Ohio, United States; University of Iowa, Iowa City, Iowa, United States; University of Iowa, IOWA CITY, Iowa, United States

## Abstract

This study aims to identify elder abuse training needs among social services directors. Using nationally representative data from the 2019 National Nursing Home Social Services Directors Survey, we found that approximately three-quarters of directors reported either a moderate or strong interest in receiving training in the following areas: (1) the types of abuse, neglect, and/or exploitation by staff, residents, family and/or other visitors…; (2) establishing trust with a resident and/or family member who feels mistreated by the nursing home or a staff member…; and (3) interacting with visitors whose behaviors are perceived as threatening to residents or staff. Hierarchical logistic regressions found significant associations between strong interest in each training topic and facility, department, and director characteristics. Additional training may enhance directors’ skills in explaining basic information about elder abuse and supporting other staff with strategies to address elder abuse and difficult interpersonal dynamics among staff, residents, families, and visitors.

